# A cross-sectional study of university students’ awareness, knowledge, and attitudes on organ donation and transplantation in Northern Cyprus

**DOI:** 10.1097/MD.0000000000038701

**Published:** 2024-06-28

**Authors:** Necmi Bayraktar, Ümmü Bayraktar

**Affiliations:** a Cyprus International University School of Medicine, Urology Department, Northern Cyprus, Nicosia; b Girne American University, Faculty of Communications, Northern Cyprus, Kyrenia.

**Keywords:** attitudes, knowledge, organ donation, organ transplantation, university students

## Abstract

**Background:**

Understanding the knowledge and attitudes of university students regarding organ transplantation and donation is crucial, as these students can significantly influence public opinion and behavior. This study aims to assess the knowledge and attitudes of North Cyprus University students towards organ transplantation and donation.

**Methods:**

A cross-sectional study was conducted with 400 students from Northern Cyprus University, divided into medical and social science faculties. A structured questionnaire was utilized to assess their knowledge and attitudes towards organ transplantation and donation. Descriptive statistics, Chi-square tests, and effect size calculations were employed for data analysis.

**Results:**

Among the 400 students, 27% demonstrated sufficient knowledge of organ transplantation, and 62.7% had positive views on organ donation. Willingness to donate was expressed by 37% as living donors and 64% as deceased donors. There were no significant differences in knowledge and attitudes between medical and social science students. Factors such as sex, marital status, faculty, and contact with individuals with end-stage organ failure did not significantly influence the knowledge and attitudes.

**Conclusions:**

This study highlights the necessity for educational interventions and awareness campaigns to improve understanding and attitudes towards organ donation among Northern Cyprus University students. Incorporating organ donation education into university curricula, providing accurate online information, addressing misconceptions, and promoting awareness of donation centers and transplant hospitals are essential steps to mitigate organ shortages. Public engagement should be encouraged to foster a supportive environment for organ donation.

## 1. Introduction

Organ transplantation is more dependable and effective in treating organ failure compared to other organ replacement therapies. Organ shortages pose the greatest challenge to global organ transplant efforts.^[1]^ Priority should be given to raising the rate of organ transplantation from deceased donors to solve organ shortage.^[2]^ Increasing organ donations is essential for advancing organ transplantation, and sociocultural structures play a crucial role in promoting both living and deceased organ transplantation.^[2,3]^ However, society’s awareness, knowledge, and attitudes toward organ transplantation are essential.

To encourage widespread support for organ donation, it’s crucial to reach out to well-informed individuals who occupy influential positions within their communities.^[4]^ By addressing their concerns and expanding their knowledge, these individuals’ perspectives could be transformed, which in turn shapes public perception.^[5]^

Based on data from the Northern Cyprus Statistical Institution, it is evident that Northern Cyprus, a nation with a population of approximately 400,000, has a thriving academic community. There are 25 universities in the country that serve more than 100,000 students. In 2016, the implementation of comprehensive legislation paved the way for the initiation of the organ transplant program, starting with living and deceased kidney donations, followed by the landmark achievement of the first heart transplant in 2019. Organ donation requests can only be submitted to the Northern Cyprus Ministry of Health. In Northern Cyprus, only heart and kidney transplants are currently performed. Previously, organ transplant surgeries were conducted at Burhan Nalbantoglu State Hospital.

The primary aim of this study was to identify the barriers and knowledge gaps that contribute to organ shortage with the goal of enhancing organ donation rates. Specifically, we focused on assessing the awareness, attitudes, and knowledge of university students regarding organ donation with and without a medical educational background. By shedding light on the perceptions and understanding of this influential group, we can uncover valuable insights that will inform targeted interventions and initiatives to address organ shortages effectively.

In this cross-sectional study, we explored the multifaceted aspects of organ transplantation in Northern Cyprus. By exploring the attitudes and knowledge of university students, we aim to contribute to the existing literature and pave the way for evidence-based strategies to promote organ donation and transplantation in the region. This study aims to close the gap between knowledge and action so that effective and sustainable strategies can be developed to boost organ transplantation rates by utilizing both accurate and inaccurate data.

## 2. Material methods

### 2.1. Structuring the study

This cross-sectional study included 400 university students enrolled between March and May of 2023. The participants were categorized into 2 distinct groups based on their academic background. The first group was comprised of students from health sciences faculties, specifically those studying nursing, physiotherapy, and paramedics. These students had received medical education. The second group consisted of students enrolled in the Faculty of Social Sciences but did not have a medical education background. By stratifying the participants into distinct groups, this study aimed to explore potential differences in their knowledge and attitudes toward organ transplantation and donation based on their academic disciplines.

### 2.2. Inclusion and exclusion criteria

Specific inclusion and exclusion criteria were used to ensure the selection of eligible participants. The inclusion criteria were University students aged ≥18 years who were enrolled in the same institution and voluntarily expressed their willingness to participate were included. The exclusion criteria were individuals below the age of 18, those holding multiple university degrees, and those who declined to participate in the study. By implementing these rigorous criteria, this study aimed to maintain a homogeneous sample and ethical standards for participant selection.

### 2.3. *Ethical* considerations

This study was approved by the Ethics Committee of Girne American University in Northern Cyprus. (Date: 31.5.2022 issue: 2021-22/011.)

### 2.4. Study design and questionnaire features

Epi Info STATCALC software was used to calculate the survey population. To arrive at a conclusive result, we utilized the following equation: n = [1962 × *P* (1 − *P*)]/α2 with a 95% confidence interval, *P* set at 0.5, a 5% margin of error, and the total student population sampled in Northern Cyprus. Following a comprehensive review of the relevant literature, a questionnaire was developed to assess the key variables of interest in this study. The questionnaire employed a Likert scale, with participants providing responses of “yes,” “no,” or “I don’t know.” It consists of 17 questions divided into 2 distinct sections: knowledge pertaining to organ transplantation and attitudes and barriers associated with organ donation and transplantation. Questions 1 to 8 assess knowledge, and questions 9 to 17 focus on attitudes. A score above 60% indicates appropriate knowledge and positive attitudes.^[5,6]^ A pilot test was conducted on a sample of 15 students to ensure the validity and clarity of the questionnaire. The internal consistency of the questionnaire was assessed using Cronbach α coefficient and a composite reliability test, both of which exceeded the acceptable threshold of 0.70. Any ambiguities or issues with the questionnaire were identified and rectified based on the feedback received during the pilot test. Importantly, the participants who took part in the pilot test were excluded from the final study sample to avoid any potential bias. This rigorous approach to questionnaire development and testing enhances the reliability and validity of the instrument, ensuring that it effectively captures the intended constructs and facilitates meaningful data collection and analysis for the objectives of the study. The questionnaire form is presented in Table 1, translated from Turkish to English.

**Table 1:** Questionnaire form.

Age:		Gender:		Date:
**Marital Status**	Married **1**	Single **2**	Widow **3**	Divorced **4**
**Do you know anyone with organ failure?**			yes **1**	no **2**
**Do you have a family member with organ failure?**			yes **1**	no **2**
**Education status?**		1- Teaching Assistant 2- Student 3- Expert **Faculties? ……………………………………………………**		
**Why Organ transplantation is important for TRNC?** **(Write in one sentence**)				
**WHERE ORGAN DONATION CAN BE MADE? (Multiple answers can be marked**)		1. Ministry of Health 2. State Hospitals 3. Private Hospitals 4. Online Application 5. Government institutions 6. Unsure 7. No Such Institution		
**Which organs are transplanted in the TRNC? (Multiple answers can be marked**)		**1** It’s not doner **2** Liver **3** Kidney **4** Pancreas **5** Heart **6** Lung **7** Cornea **8** Small intestines		
**In which hospitals organ transplantation is performed in TRNC? (Multiple answers can be marked**)		**1** Not done **2** Kyrenia Akcicek Hospital **3** Cengiz Topel Hospital **4** Burhan Nalbantoglu State Hospital **5** Near East University Hospital **6** Magosa State Hospital **7** Magosa Medical Center Hospital **8** Kolan British Hospital **9** University of Kyrenia Hospital		

Please answer the questions as Yes No I don't know. (X)		Yes	No	IDK
**1. Did you know that organ transplants are performed in Northern Cyprus??**		1	2	3
**2. “Is it legal to undergo organ transplant procedures in Northern Cyprus?”**		1	2	3
**3. Is Organ Donation Possible After Death?**		1	2	3
**4. Is there availability of qualified human resources and equipment for organ transplantation in Northern Cyprus?**		1	2	3
**5. Many countries accept organ donation after death?**		1	2	3
**6. Is organ donation after brain death considered legal in Northern Cyprus?**		1	2	3
**7. Do You Know That Organ Donation Can Be Done in Countries Near Us After Brain Death?**		1	2	3
**8. Do You Have Any Knowledge About What the Brain Death Condition Is?**		1	2	3
**9. Would You Like to Be a Kidney or Liver Organ Donor in Your Life?**		1	2	3
**10. Would You Agree to Donate Your Organs After Death?**		1	2	3
**11. If you have end-stage renal failure, would you agree to receive a kidney transplant from a deceased donor?**		1	2	3
**12. Do You Rely on the Health Care System to Identify and Diagnose Brain Death?**		1	2	3
**13. Would You Encourage Organ Donors or Their Families to Donate After Brain Death?**		1	2	3
**14. Do you believe that brain death constitutes irreversible death?**		1	2	3
**15. Do You Consider the Lack of Renal Donation in End-Stage Renal Failure to Be a Health Concern?**		1	2	3
**16. Do you believe that organ donation and transplant awareness should be included in the school curriculum?**		1	2	3
**17. Should Organ Donations and Program Policies Be Conducted by the Government?**		1	2	3
**18. How did you get the information that determined your answers?**		**(1) Lectures** **(2) Radio-Television** **(3) Online Sources and Social Networks** **(4) Newspapers** **(5) Family and Friends** **(6) Campaigns** **(7) Seminar and Conference**		

### 2.5. Data analysis

Frequencies and percentages were used to represent categorical data such as gender, age, marital status, and faculty. Continuous data are presented as the mean and standard deviation. The normality of distributions was examined using the Kolmogorov–Smirnov and Shapiro–Wilk tests. An independent sample Chi-square test was performed to measure the significant relationship between sociodemographic data and knowledge of and attitudes toward organ donation. A *P* value ≤.05 was considered statistically significant. The *P* value was interpreted by calculating the effect size. Cohen benchmarks suggest using the terms small (*d* = 0.2), medium (*d* = 0.5), and large (*d* = 0.8) to describe effect sizes. This is a widely accepted way of categorizing effect sizes.^[7]^ Data analysis was conducted using IBM SPSS Statistics for Windows, Version 28.0. Armonk, NY: IBM Corp.

## 3. Results

A total of 400 students participated in the study. Among them, 221 (55.3%) were female, and 179 (44.8%) were male. The mean age of the patients was 22 ± 3.46 years. Most participants, 373 (93.3%), were single, 24 (6%) were married, and 3 (0.8%) were divorced or widowed. Among participants, 53 (13%) reported being acquainted with end-stage organ failure. Additionally, 27 individuals (6.8%) stated that they had a family member with end-stage organ failure.

Regarding the question, “Why is organ transplantation important for Northern Cyprus?” A total of 103 (25.8%) respondents did not provide an answer, 154 (38.5%) answered that it was to save lives, 55 (13%) stated that it was to help patients, 40 (10%) mentioned organ shortages, and 48 (12%) indicated prestige as a reason. The data exhibited a normal distribution, and no statistically significant difference was observed between the 2 groups (*P* = .25). However, when calculating the effect size (Cohen *d*), it was found to be −0.224, with a 95% confidence interval ranging from −0.421 to −0.028.

Students’ characteristics and attitudes toward organ donation and transplantation were further analyzed based on their faculties, and the results are presented in Table 2. The majority (30.7%) of the students who completed the questionnaire reported obtaining their knowledge from online sources and social networks. Specifically, 135 participants from the Faculty of Health Sciences stated that they responded based on the information obtained from lectures, whereas 152 participants from the Faculty of Social Sciences relied on online resources and social networks. A detailed summary of the categories of the faculty members is presented in Table 3. To the question of “where to apply for organ donation in Northern Cyprus?” A total of 202 (50.5%) replied to the Ministry of Health, 201 (50.2%) to State Hospitals, and 104 (26%) as I do not know. The answers given by the students according to the faculty are shown in Table 4. In response to the query pertaining to the organ transplants conducted in Northern Cyprus, 188 participants (47.1%) opined that no organ transplants are performed in Northern Cyprus, whereas kidney and heart transplants can be carried out. Furthermore, the remaining responses indicated that transplants of organs that cannot be performed can also be performed. According to the faculty, “What organ transplants are being done in Northern Cyprus?” The answers to the questions in Table 5 are also presented. According to the survey, 139 (34.8%) participants answered the Near East University Hospital, and 129 (32.3%) said that organ transplantation was performed at the Burhan Nalbantoglu State Hospital. The responses of the organ transplant hospitals according to the faculty of the participants are presented in Table 6.

**Table 2:** Characteristics of the students toward organ donation and transplantation.

Characteristics	Faculty of Social Sciences, N (%)	Faculty of Health Sciences, N (%)
Gender		
Male	94 (47%)	84 (42%)
Female	106 (53%)	116 (58%)
Age (mean ± SD)	23.2 ± 6.4	21.5 ± 4.3
Marital status		
** **Single	181 (90.5%)	192 (96%)
** **Married	17 (8.5%)	7 (3.5%)
** **Divorced/widow	2 (%1)	1 (0.5%)
SRWO		
** **Yes	18 (9%)	9 (4.5%)
** **No	182 (91%)	191 (95.5%)
SWKSWO		
** **Yes	27 (13.5%)	25 (12.5%)
** **No	172 (86%)	175 (87.5%)
IOTNC		
** **No answer	53 (26.5%)	50 (25%)
** **Saving life	59 (29.5%)	95 (47.5%)
** **Helping a patient	37 (18.5%)	18 (9%)
** **Organ shortage	20 (10%)	20 (10%)
** **Prestige	31 (15.5%)	17 (8.5%)

IOTNC = the importance of organ transplant for Northern Cyprus, SD = standard deviation, SRWO = student relatives waiting for organs, SWKSWO = students who know someone waiting for an organ.

**Table 3:** Sources of knowledge about organ donation among students.*

Faculty of Health Sciences	N	Faculty of Social Sciences	N
Lectures	135	Online sources and social networks	152
Online sources and social networks	101	Family and friends	81
Family and friends	47	Lectures	81
Radio-television	29	Radio-television	60
Seminar and conference	24	Newspapers	32
Newspapers	18	Campaigns	26
Campaigns	18	Seminar and conference	20
Total†	372	Total	452

N = number.

* Multiple answer survey question.

† Total number of responses in the relevant faculty.

**Table 4:** Where to donate organs in Northern Cyprus according to participants?*

		Number of responses	Percentage of responses from each faculty
Faculty of Health Sciences	Ministry of Health	100	26.4% (50.0%)
	State hospitals	111	29.3% (55.5%)
	Private hospitals	56	14.8% (28.0%)
	Online application	35	9.2% (17.5%)
	Government institutions	22	5.8% (11.0%)
	Unsure	54	14.2% (27.0%)
	No such institution	1	0.3% (0.5%)
Faculty of Social Sciences	Ministry of Health	102	28.6% (51.0%)
	State hospitals	90	25.2% (45.0%)
	Private hospitals	47	13.2% (23.5%)
	Online application	38	10.6% (19.0%)
	Government institutions	13	3.6% (6.5%)
	Unsure	50	14.0% (25.0%)
	No such institution	17	4.8% (8.5%)

* Multiple answer survey question.

**Table 5:** According to the participants, which organs are transplanted in Northern Cyprus?*

Faculties		Responses	
		N	Percent
Faculty of Health Sciences	Not being done	91	22.9%
	Liver	54	13.6%
	Kidney	89	22.4%
	Pancreas	21	5.3%
	Heart	65	16.4%
	Lung	35	8.8%
	Cornea	25	6.3%
	Small intestine	17	4.3%
	Total	397	100.0%
Faculty of Social Sciences	Not being done	97	28.0%
	Liver	46	13.3%
	Kidney	83	24.0%
	Pancreas	16	4.6%
	Heart	56	16.2%
	Lung	28	8.1%
	Cornea	15	4.3%
	Small intestine	5	1.4%
	Total	346	100.0%

N = number.

* Multiple answer survey question.

**Table 6:** Which hospitals in Northern Cyprus perform organ transplants according to the participants?*

Faculties	Hospital	Responses	
		N	Percent
Faculty of Health Sciences	Not being done	91	37.6%
	Nalbantoglu State Hospital	66	27.3%
	NEU Hospital	65	26.9%
	Famagusta State Hospital	1	0.4%
	Kolan British Hospital	1	0.4%
	Kyrenia University Hospital	18	7.4%
	Total	242	100.0%
Faculty of Social Sciences	Not being done	97	36.3%
	Akçiçek State Hospital	11	4.1%
	Cengiz Topel State Hospital	2	0.7%
	Nalbantoglu State Hospital	63	23.6%
	NEU Hospital	74	27.7%
	Famagusta State Hospital	2	0.7%
	Kolan British Hospital	2	0.7%
	Kyrenia University Hospital	16	6.0%
	Total	267	100.0%

NEU Hospital = Near East University Hospital.

* Multiple answer survey question.

Based on the statistical analysis, factors such as sex, marital status, or having a family member or acquaintance with end-stage organ failure do not seem to affect one’s level of knowledge. To evaluate knowledge level, questions 1 to 8 were assessed, with scores ≥60% considered satisfactory level of knowledge. However, only a small percentage of students (27%) demonstrated good knowledge. Independent sample *t* tests revealed no significant difference in knowledge levels between the 2 groups based on faculty, with a small effect size (*P* = .22, Cohen *d* effect size of 0.07, 95% confidence interval between −0.120 and −0.272). To assess the level of attitude, questions 9 to 17 were evaluated, and participants who scored ≥60% were considered to have a positive attitude. It was determined that 62.7% of the participants had positive attitudes. The data exhibited a normal distribution. When students’ attitudes were compared according to their faculties, it was found that the faculty of social sciences had a higher attitude level based on parametric analysis (independent sample *t* test), and a statistically significant difference was observed between the 2 groups (*P* = .02). However, the effect size was less significant than Cohen *d* (point estimate: 0.309, 95% confidence interval = 0.112–0.506). Table 7 provides an in-depth analysis of the responses provided by students regarding their knowledge and attitude levels toward organ donation.

**Table 7:** Students’ responses to knowledge and attitude levels about organ donation.

	Faculty of Health Sciences			Faculty of Social Sciences		
	Yes	No	IDK	Yes	No	IDK
	N %	N %	N %	N %	N %	N %
1. Did you know that organ transplants are performed in Northern Cyprus?	44 (22.0%)	50 (25.0%)	**106 (53.0%**)	56 (28.0%)	40 (20.0%)	**104 (52.0%**)
2 “Is it legal to undergo organ transplant procedures in Northern Cyprus?”	69 (34.5%)	10 (5.0%)	**121 (60.5%**)	77 (38.5%)	5 (2.5%)	**118 (59.0%**)
3. Is organ donation possible after death?	**149 (74.5%**)	25 (12.5%)	26 (13.0%)	**138 (69.0%**)	25 (12.5%)	37 (18.5%)
4. Is there availability of qualified human resources and equipment for organ transplantation in Northern Cyprus?	29 (14.5%)	28 (14.0%)	**143 (71.5%**)	32 (16.0%)	34 (17.0%)	**134 (67.0%**)
5. Many countries accept organ donation after death?	**93 (46.5%**)	30 (15.0%)	77 (38.5%)	**124 (62.0%**)	15 (7.5%)	61 (30.5%)
6. Is organ donation after brain death considered legal in Northern Cyprus?	58 (29.0%)	15 (7.5%)	**127 (63.5%**)	45 (22.5%)	15 (7.5%)	**140 (70.0%**)
7. Do you know that organ donation can be done in countries near US after brain death?	66 (33.0%)	39 (19.5%)	**95 (47.5%**)	76 (38.0%)	25 (12.5%)	**99 (49.5%**)
8. Do you have any knowledge about what the brain death condition is?	**130 (65.0%**)	29 (14.5%)	41 (20.5%)	**142 (71.0%**)	20 (10.0%)	38 (19.0%)
9. Would you like to be a kidney or liver organ donor in your life?	**77 (38.5%**)	55 (27.5%)	68 (34.0%)	**73 (36.5%**)	60 (30.0%)	67 (33.5%)
10. Would you agree to donate your organs after death?	**119 (59.5%**)	30 (15.0%)	51 (25.5%)	**137 (68.5%**)	25 (12.5%)	38 (19.0%)
11. If you have end-stage renal failure, would you agree to receive a kidney transplant from a deceased donor?	**91 (45.5%**)	55 (27.5%)	54 (27.0%)	**113 (56.5%**)	36 (18.0%)	51 (25.5%)
12. Do you rely on the health care system to identify and diagnose brain death?	**87 (43.5%**)	41 (20.5%)	72 (36.0%)	65 (32.5%)	**75 (37.5%**)	60 (30.0%)
13. Would you encourage organ donors or their families to donate after brain death?	**105 (52.5%**)	34 (17.0%)	61 (30.5%)	**125 (62.5%**)	27 (13.5%)	48 (24.0%)
14. Do you believe that brain death constitutes irreversible death?	**116 (58.0%**)	28 (14.0%)	56 (28.0%)	**154 (77.0%**)	19 (9.5%)	27 (13.5%)
15. Do you consider the lack of renal donation in end-stage renal failure to be a health concern?	**113 (56.5%**)	23 (11.5%)	64 (32.0%)	**138 (69.0%**)	24 (12.0%)	38 (19.0%)
16. Do you believe that organ donation and transplant awareness should be included in the school curriculum?	**119 (59.5%**)	27 (13.5%)	54 (27.0%)	**141 (70.5%**)	31 (15.5%)	28 (14.0%)
17. Should organ donations and program policies be conducted by the government?	**122 (61.0%**)	26 (13.0%)	52 (26.0%)	**149 (74.5%**)	14 (7.0%)	37 (18.5%)

Bold lines indicate the majority answer.

IDK = I don’t know, N = number.

## 4. Discussion

This study aimed to evaluate the knowledge and attitudes of university students in Northern Cyprus regarding organ transplantation and donation. The findings offer valuable insights into the understanding and perceptions of young individuals, who play a pivotal role in shaping public opinion and behaviors toward organ donation. In general, the results indicate that there is a need for improvement in both knowledge and attitudes toward organ transplantation and donation among university students in Northern Cyprus. The responses to the question “Why is organ transplantation important for Northern Cyprus?” revealed that the majority of individuals either did not have information about the issue or did not provide an answer. Furthermore, it appears that a substantial number of respondents were unaware of the critical aspects of organ shortage. Moreover, stereotypical slogans such as “saving lives” and “helping patients” have been emphasized.^[8]^ However, the importance of organ transplantation for every country is that there is a shortage of organs. Therefore, answering such an important question with stereotypical slogans not only shows a lack of knowledge but also highlights the importance of previous information and awareness campaigns. This underscores the necessity for enhanced education and awareness campaigns to bridge the knowledge gap and enhance understanding of the importance of organ transplantation.

Interestingly, this study also found a disparity in attitudes between medically educated students and students from nonmedical backgrounds. Although the effect size was not significant, we believe that it was influenced by the effect of education. It is necessary to create an educational reflex to address organ shortages, such as by protecting the environment and preserving natural resources. This finding highlights the potential impact of educational background on shaping attitudes and the importance of incorporating organ donation and transplant education into the curricula of different disciplines. Because organ transplantation is the most effective treatment for damaged organs, it is critical to raise awareness and understanding of it.

Furthermore, this study sheds light on the sources of information regarding organ transplantation and donation. Many students rely on online sources and social networks to acquire knowledge (30.7%). This finding is consistent with those in the literature.^[9,10]^ Moreover, research has shown that family and immediate surroundings play crucial roles in shaping attitudes and acquiring knowledge. They are often considered primary sources of knowledge. This underscores the need for readily accessible, accurate, and reliable online information, as well as the significance of utilizing social media platforms to disseminate educational content about organ transplantation and donation.

A notable finding from the study is that the participants displayed a deficiency of information regarding the places where organ donation can be made and the hospitals that carry out organ transplantation in Northern Cyprus. The survey results indicate this lack of knowledge, as the answers provided regarding the institutions where the majority of organ transplants are performed reveal the participants’ lack of information. Although the hospitals have not made any formal statements or announcements about organ transplantation, the institution’s numerous advertisements and campaigns promoting technological advancements and innovations may have contributed to the perception that they do perform organ transplants. However, in reality, no organ transplants have taken place, except for emphasizing the importance of organ transplantation and donation. This observation highlights the influence of advertising and promotions on public expectations, as the participants’ responses reflect their expectations based on their perceptions rather than reality. It is worth noting that the lack of information sharing and publicity about a hospital that has been performing organ transplantation for 7 years is another issue that needs to be addressed. Communicating successful work to the public through the appropriate channels of information is just as important as the work itself. The participants’ lack of awareness of the channels available for organ donation highlights the need for better information dissemination and improved communication strategies by the relevant authorities.^[11]^ Efforts should be made to ensure that individuals have easy access to information regarding organ donation centers and associated procedures.^[12–15]^

Our findings are consistent with those of a recent poll conducted in Japan,^[16]^ indicating a similar discrepancy between the willingness to donate organs and the actual declaration of intention among university students in Northern Cyprus. While positive attitudes toward organ donation were prevalent, the proportion of students who expressed their intention to donate was significantly lower. This highlights the need for further research on the factors influencing the intention-action gap and the implementation of targeted interventions to promote organ donation. Emphasizing the importance of public participation and awareness campaigns is crucial to address misconceptions and expand the pool of organ donors.

Overall, this study emphasizes the significance of educational initiatives and awareness campaigns targeting university students in Northern Cyprus to enhance their knowledge and attitudes toward organ transplantation and donation. Implementation of educational programs, both within health sciences faculties and across other disciplines, can contribute to raising awareness and fostering a positive attitude toward organ donation.^[6,17,18]^ The state-led initiatives, including information, campaigns, and awareness activities, will help to prevent misinformation and provide easy access to accurate information and messages. This will ensure that individuals who work and contribute to the field are justly remunerated, fostering an environment of trust for society and future generations. Our research recommends that actions should be taken to ensure that verifiable and dependable information is readily available both on the internet and social media platforms.

### 4.1. Study limitations

It is important to note that this study has certain limitations. A cross-sectional design restricts the ability to establish causality or to assess changes in attitudes over time. The study sample was comprised of university students from a specific region, which may limit the generalizability of our findings to other populations. The fact that students from different universities and faculty members were not included in the study remains a limiting factor, even if the sample size was sufficient. Future research should involve a larger and more diverse sample to provide a more comprehensive understanding of the knowledge and attitudes toward organ transplantation and donation among university students in different regions.

## 5. Conclusion

The results highlight the necessity for targeted educational interventions and awareness campaigns to improve knowledge, correct misconceptions, and foster positive attitudes toward organ donation. Providing accurate information to young people can help create a supportive environment for organ transplantation.

## Author contributions

**Conceptualization:** Necmi Bayraktar, Ümmü Bayraktar.

**Data curation:** Necmi Bayraktar, Ümmü Bayraktar.

**Formal analysis:** Necmi Bayraktar, Ümmü Bayraktar.

**Funding acquisition:** Necmi Bayraktar, Ümmü Bayraktar.

**Investigation:** Necmi Bayraktar, Ümmü Bayraktar.

**Methodology:** Necmi Bayraktar, Ümmü Bayraktar.

**Project administration:** Necmi Bayraktar, Ümmü Bayraktar.

**Resources:** Necmi Bayraktar, Ümmü Bayraktar.

**Software:** Necmi Bayraktar, Ümmü Bayraktar.

**Supervision:** Necmi Bayraktar, Ümmü Bayraktar.

**Validation:** Necmi Bayraktar, Ümmü Bayraktar.

**Visualization:** Necmi Bayraktar, Ümmü Bayraktar.

**Writing—original draft:** Necmi Bayraktar, Ümmü Bayraktar.

**Writing—review & editing:** Necmi Bayraktar, Ümmü Bayraktar.

## References

[1] Stephan A. Organ shortage: can we decrease the demand? Exp Clin Transplant. 2017;15:6–9. 28260423 10.6002/ect.mesot2016.L27

[2] Tonkens R. The most good you can do with your kidneys: effective altruism and the organ-shortage problem. J Med Philos. 2021;46:350–76. 34106278 10.1093/jmp/jhab007

[3] Sterri AB Regmi S Harris J. Ethical solutions to the problem of organ shortage. Camb Q Healthc Ethics. 2022;31:297–309. 35899548 10.1017/S0963180121000955

[4] Salmani Nadoushan M Nozary Heshmati B Shabanzadeh Pirsaraee A Salmani Nodoushan I Jafari Nadoushan R Yazdi F. Knowledge and attitude of Iranian physicians towards organ and tissue donation. Int J Organ Transplant Med. 2014;5:66–70. 25013681 PMC4089334

[5] Asimakopoulou E Stylianou V Dimitrakopoulos I Argyriadis A Bellou-Mylona P. Knowledge and attitudes regarding organ transplantation among Cyprus residents. J Nurs Res. 2020;29:e132. 33156139 10.1097/jnr.0000000000000409 PMC7808348

[6] Alwahaibi N Al Wahaibi A Al Abri M. Knowledge and attitude about organ donation and transplantation among Omani university students. Front Public Health. 2023;11:1115531. 37304098 10.3389/fpubh.2023.1115531 PMC10248022

[7] Cohen J. Statistical power analysis for the behavioral sciences. Routledge: Academic Press; 2013.

[8] Green M Byrne MHV Legard C. The effect of positive and negative poster messages on organ donor registration. Transplant Proc. 2020;52:2899–900. 32430146 10.1016/j.transproceed.2020.03.029

[9] Majeed F. Saudi nursing and medical student’s knowledge and attitude toward organ donation-a comparative cross-sectional study. Int J Health Sci (Qassim). 2016;10:209–17. 27103903 PMC4825894

[10] Uzuntarla Y. Knowledge and attitudes of health personnel about organ donation: a tertiary hospital example, Turkey. Transplant Proc. 2018;50:2953–60. 30577154 10.1016/j.transproceed.2018.08.004

[11] Stevens J Tumin D Shaffer KL Bickman L Hoagwood KE Hayes D Jr. Are there missed opportunities to maximize organ donation registrations? An examination of driver’s license applications across the United States. Prog Transplant. 2019;29:173–8. 30845877 10.1177/1526924819835832

[12] Kumar K King EA Muzaale AD. A smartphone app for increasing live organ donation. Am J Transplant. 2016;16:3548–53. 27402293 10.1111/ajt.13961

[13] Baan CC Dor FJ. The transplantation journal on social media: the @TransplantJrnl journey from impact factor to Klout Score. Transplantation. 2017;101:8–10. 27906823 10.1097/TP.0000000000001581

[14] Henderson ML Adler JT Van Pilsum Rasmussen SE. How should social media be used in transplantation? A survey of the American Society of Transplant Surgeons. Transplantation. 2019;103:573–80. 29684002 10.1097/TP.0000000000002243 PMC6196114

[15] Sallis A Harper H Sanders M. Effect of persuasive messages on National Health Service Organ Donor Registrations: a pragmatic quasi-randomised controlled trial with one million UK road taxpayers. Trials. 2018;19:513. 30241564 10.1186/s13063-018-2855-5 PMC6150960

[16] Hirai K Ohtake F Kudo T. Effect of different types of messages on readiness to indicate willingness to register for organ donation during driver’s license renewal in Japan. Transplantation. 2020;104:2591–8. 32058465 10.1097/TP.0000000000003181

[17] Bedi KK Hakeem AR Dave R Lewington A Sanfey H Ahmad N. Survey of the knowledge, perception, and attitude of medical students at the University of Leeds toward organ donation and transplantation. Transplant Proc. 2015;47:247–60. 25769557 10.1016/j.transproceed.2014.11.033

[18] Iniesta-Sepúlveda M López-Navas AI Gutiérrez PR Ramírez P Ríos A. The willingness to donate organs in medical students from an international perspective: a meta-analysis. Transpl Int. 2022;35:10446. 35837470 10.3389/ti.2022.10446 PMC9273723

